# COVID-19–Associated Hospitalizations and Maternal Vaccination Among Infants Aged <6 Months — COVID-NET, 12 States, October 2022–April 2024

**DOI:** 10.15585/mmwr.mm7338a1

**Published:** 2024-09-26

**Authors:** Fiona P. Havers, Michael Whitaker, Bhoomija Chatwani, Monica E. Patton, Christopher A. Taylor, Shua J. Chai, Breanna Kawasaki, Kimberly Yousey-Hindes, Kyle P. Openo, Patricia A. Ryan, Lauren Leegwater, Ruth Lynfield, Daniel M. Sosin, Bridget J. Anderson, Brenda Tesini, Melissa Sutton, H. Keipp Talbot, Andrea George, Jennifer Milucky, COVID-NET Surveillance Team, COVID-NET Surveillance Team, Darpun Sachdev, Nisha Alden, Emily Zmek, Lucy Witt, Maya L. Monroe, Elizabeth McCormick, Paige D'Heilly, Susan L. Ropp, Kerianne Engesser, Erin Licherdell, Sam Hawkins, William Schaffner, Holly Staten

**Affiliations:** ^1^Coronavirus and Other Respiratory Viruses Division, National Center for Immunization and Respiratory Diseases, CDC; ^2^Eagle Health Analytics, Atlanta, Georgia; ^3^California Emerging Infections Program, Oakland, California; ^4^Career Epidemiology Field Officer Program, CDC; ^5^Colorado Department of Public Health & Environment; ^6^Connecticut Emerging Infections Program, Yale School of Public Health, New Haven, Connecticut; ^7^Emory University School of Medicine, Atlanta, Georgia; ^8^Georgia Emerging Infections Program, Georgia Department of Public Health; ^9^Atlanta Veterans Affairs Medical Center, Decatur, Georgia; ^10^Maryland Department of Health; ^11^Michigan Department of Health and Human Services; ^12^Minnesota Department of Health; ^13^New Mexico Department of Health; ^14^New York State Department of Health; ^15^University of Rochester School of Medicine and Dentistry, Rochester, New York; ^16^Public Health Division, Oregon Health Authority; ^17^Vanderbilt University Medical Center, Nashville, Tennessee; ^18^Salt Lake County Health Department, Salt Lake City, Utah.; California Department of Public Health; Colorado Department of Public Health & Environment; Connecticut Emerging Infections Program, Yale School of Public Health; Division of Infectious Diseases,; Emory University of Medicine, Georgia Emerging Infections Program; Maryland Department of Health; Michigan Department of Health and Human Services; Minnesota Department of Health; New Mexico Department of Health; New York State Department of Health; University of Rochester School of Medicine and Dentistry; Public Health Division, Oregon Health Authority; Vanderbilt University Medical Center; Salt Lake County Health Department.

**Summary**

**What is already known about this topic?**

Infants aged <6 months have high COVID-19–associated hospitalization rates and are not age-eligible for COVID-19 vaccination.

**What is added by this report?**

COVID-19–associated hospitalization rates among infants aged <6 months remain higher than those among any other age group except adults aged ≥75 years and were comparable to hospitalization rates in adults aged 65–74 years. Among approximately 1,000 hospitalized infants with COVID-19, 22% were admitted to an intensive care unit, and nine died while hospitalized. The percentage of hospitalized infants whose mothers had been vaccinated during pregnancy was 18% during October 2022–September 2023 and decreased to <5% during October 2023–April 2024.

**What are the implications for public health practice?**

COVID-19 can cause severe disease in infants aged <6 months; prevention should focus on ensuring that pregnant persons receive recommended COVID-19 vaccines to protect themselves and their young infants.

## Abstract

Infants aged <6 months are at increased risk for severe COVID-19 disease but are not yet eligible for COVID-19 vaccination; these children depend upon transplacental transfer of maternal antibody, either from vaccination or infection, for protection. COVID-19–Associated Hospitalization Surveillance Network (COVID-NET) data were analyzed to estimate COVID-19–associated hospitalization rates and identify demographic and clinical characteristics and maternal vaccination status of infants aged <6 months hospitalized with laboratory-confirmed COVID-19. During October 2022–April 2024, COVID-NET identified 1,470 COVID-19–associated hospitalizations among infants aged <6 months. COVID-19–associated hospitalization rates among young infants were higher than rates among any other age group, except adults aged ≥75 years, and are comparable to rates among adults aged 65–74 years. The percentage of hospitalized infants whose mothers had been vaccinated during pregnancy was 18% during October 2022–September 2023 and decreased to <5% during October 2023–April 2024. Severe outcomes among infants hospitalized with COVID-19 occurred frequently: excluding newborns hospitalized at birth, approximately one in five young infants hospitalized with COVID-19 required admission to an intensive care unit, nearly one in 20 required mechanical ventilation, and nine infants died during their COVID-19–associated hospitalization. To help protect pregnant persons and infants too young to be vaccinated, prevention for these groups should focus on ensuring that pregnant persons receive recommended COVID-19 vaccines.

## Introduction

COVID-19 can cause severe disease in children, and infants aged <6 months have the highest COVID-19 hospitalization rates among all pediatric age groups (*1*,*2*). These infants are not yet age-eligible to receive COVID-19 vaccination, and maternal vaccination during pregnancy protects young infants from COVID-19–associated hospitalization (*3*–*5*). Data from the COVID-19–Associated Hospitalization Surveillance Network (COVID-NET) during October 2022–April 2024 were analyzed to describe hospitalization rates, maternal vaccination status, clinical outcomes, and codetections of other viruses among infants aged <6 months hospitalized with laboratory-confirmed SARS-CoV-2 infection.

## Methods

### Data Sources

COVID-NET conducts population-based surveillance for laboratory-confirmed COVID-19–associated hospitalization, defined as documentation of a positive SARS-CoV-2 test result during hospitalization or ≤14 days preceding hospital admission, among residents of a predefined catchment area. Demographic data were collected for all COVID-19–associated hospitalizations in 90 counties across 12 states* and were used to calculate age-stratified hospitalization rates.

This analysis describes weekly and seasonal cumulative hospitalization rates among infants aged <6 months (young infants) hospitalized across two respiratory virus seasons (October 2022–September 2023 [2022–23] and October 2023–April 2024 [2023–24]).^†^ Unadjusted weekly COVID-19–associated hospitalization rates (hospitalizations per 100,000 population)^§^ with rate ratios (RRs) were calculated by dividing the total number of hospitalized patients by age group–specific population estimates for the counties included in the surveillance catchment area. When comparing cumulative rates among infants across seasons, the period was limited to October–April for both periods for comparability. When comparing rates between age groups, cumulative and weekly data comparing pediatric age groups are presented for 2022–2024; cumulative data comparing all age groups are presented for 2023–24 only.

Using previously described methods (*6*), clinical data (signs and symptoms at admission,^¶^ underlying medical conditions,** viral codetections, and clinical outcomes) were abstracted for a random sample of hospitalized infants.^††^ Maternal vaccination, defined as receipt of any COVID-19 vaccination at any time during pregnancy, was obtained through state immunization information systems; infant characteristics were compared by maternal vaccination status. The analysis of clinical data excluded newborns who received a positive SARS-CoV-2 test result during their birth hospitalization^§§^ because the clinical significance of a positive test result in this setting is difficult to determine.

### Statistical Methods

Wilcoxon rank-sum tests and Fisher’s exact chi-square tests were used to compare medians and proportions, respectively; p-values <0.05 were considered statistically significant. Percentages were weighted to account for the probability of selection for sampled cases, and further adjusted to account for nonresponse (i.e., an incomplete medical chart review). Variances were estimated using Taylor series linearization method. Data were analyzed using SAS (version 9.4; SAS Institute). This activity was reviewed by CDC, deemed not research, and was conducted consistent with applicable federal law and CDC policy.^¶¶^

## Results

### COVID-19–Associated Hospitalization Rates

**FIGURE 1:**
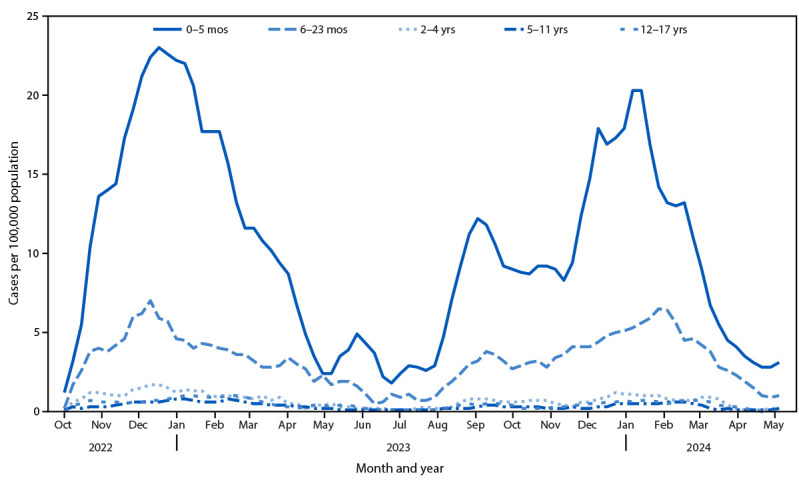
Weekly COVID-19–associated hospitalization rates (3-week moving average) among children and adolescents aged <18 years,* by age group — COVID-19–Associated Hospitalization Surveillance Network, 12 states,^†^ October 1, 2022–April 30, 2024 * Number of patients with a laboratory-confirmed COVID-19–associated hospitalization per 100,000 population. ^†^ Selected counties in California, Colorado, Connecticut, Georgia, Maryland, Michigan, Minnesota, New Mexico, New York, Oregon, Tennessee, and Utah.

During October 2022–April 2024, a total of 1,470 COVID-19–associated hospitalizations among infants aged <6 months who received a positive SARS-CoV-2 test result were identified. Weekly COVID-19 hospitalization rates were highest among infants aged <6 months compared with rates in other pediatric age groups, peaking at 23.0 and 20.3 per 100,000 infants during the weeks ending December 17 in 2022–23 and January 13 in 2023–24, respectively (Figure 1). Cumulative hospitalization rates among young infants were lower during October 2023–April 2024 (319.8) than during October 2022–April 2023 (413.6) (RR = 0.77; 95% CI = 0.69–0.86). In 2023–24, compared with rates among young infants (319.8), hospitalization rates were higher only among adults aged ≥75 years (940.1) (RR = 0.34; 95% CI = 0.31–0.37) and were comparable to hospitalization rates among adults aged 65–74 years (284.2) (RR = 1.1; 95% CI = 1.0–1.2) (Supplementary Figure, https://stacks.cdc.gov/view/cdc/162443).

Full chart reviews were conducted for a random sample of 1,266 (86%) of 1,470 hospitalized infants; among these, 118 (9.4%) were newborns who received a positive SARS-CoV-2 test result during their birth hospitalization and were excluded from the analysis of clinical data. Among these 118 infants who received a positive test result during the birth hospitalization, 23 (18.3%) had COVID-19–related signs and symptoms recorded, compared with 1,071 (92.6%) of 1,148 infants hospitalized with COVID-19 during a nonbirth hospitalization (Supplementary Table 1, https://stacks.cdc.gov/view/cdc/162444).

### Maternal COVID-19 Vaccination Status

**FIGURE 2:**
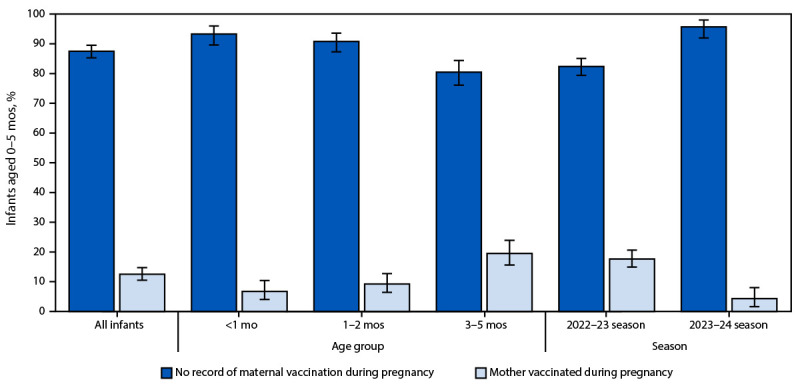
Maternal vaccination status among infants aged <6 months hospitalized with laboratory-confirmed SARS-CoV-2 infection,*^,^^†^ by age group and season^§^ — COVID-19–Associated Hospitalization Surveillance Network, 12 states,^¶^ October 2022–April 2024 * Excluding birth hospitalizations. A birth hospitalization was defined as the hospitalization during which the infant was born. ^†^ With 95% CIs indicated by error bars. ^§^ The 2022–23 season is defined as occurring during October 1, 2022–September 30, 2023, and the 2023–24 season is defined as occurring during October 1, 2023–April 30, 2024. ^¶^ Selected counties in California, Colorado, Connecticut, Georgia, Maryland, Michigan, Minnesota, New Mexico, New York, Oregon, Tennessee, and Utah.

Among 1,148 infants for whom the clinical course was assessed (Table), maternal vaccination status was available for 1,065 (92.6%). Among these infants, the mothers of 921 (87.5%) had no documentation of receipt of COVID-19 vaccination during pregnancy. The percentage of hospitalized infants whose mothers were vaccinated significantly decreased from 17.6% (132 of 745) during 2022–23 to 4.3% (12 of 320) during 2023–24 (p<0.001) (Figure 2). Infants whose mothers were vaccinated during pregnancy were older at hospitalization (median age = 109 days; IQR = 56–145 days) than were infants whose mothers had no record of vaccination during pregnancy (median age = 58 days; IQR = 28–114 days) (Supplementary Table 2, https://stacks.cdc.gov/view/cdc/162445). Among infants whose mothers’ COVID-19 vaccination status was known, all who died in-hospital were born to mothers with no record of vaccination during pregnancy.

**TABLE:** Demographic characteristics, clinical outcomes, and maternal vaccination status among infants aged <6 months hospitalized with laboratory-confirmed SARS-CoV-2 infection* — COVID-19–Associated Hospitalization Surveillance Network, 12 states,^†^ October 2022–April 2024

Characteristic	No.	Weighted %^§^ (95% CI)
**Age**		
Median age, days (IQR)	1,148	64 (28–121)
<1 mos	292	26.4 (23.5–29.5)
1–2 mos	404	34.5 (31.5–37.7)
3–5 mos	452	39.0 (35.9–42.3)
**Sex**		
Female	454	40.6 (37.3–43.9)
Male	694	59.4 (56.1–62.7)
**Race and ethnicity** ^¶^		
Asian or Native Hawaiian or Pacific Islander	65	5.7 (4.2–7.4)
Black or African American	186	16.6 (14.1–19.3)
White	408	34.6 (31.6–37.8)
Hispanic or Latino	378	33.2 (30.1–36.4)
Other races	42	3.6 (2.5–5.0)
Unknown	69	6.3 (4.8–8.0)
**Underlying conditions**		
None	858	76.0 (73.2–78.6)
One or more underlying medical condition**	290	24.0 (21.4–26.8)
Prematurity (gestational age <37 wks)	196	17.1 (14.7–19.7)
Cardiovascular disease (including congenital heart disease)	74	6.6 (5.1–8.4)
Chronic lung disease^††^	58	4.7 (3.6–6.2)
Neurologic disorders	35	3.3 (2.2–4.9)
Feeding tube dependence	30	2.4 (1.6–3.5)
**COVID-19–related signs and symptoms at admission** ^§§^	1,071	92.6 (90.7–94.2)
**Hospitalization intervention/Outcome**		
Length of hospital stay, days, median (IQR)	1,148	2 (1–3)
ICU admission	260	22.1 (19.5–24.8)
In-hospital death	9	0.8 (0.4–1.5)
**Highest level of oxygen support received during hospitalization**		
Supplemental oxygen	251	23.0 (20.1–26.1)
High flow nasal canula or BiPAP/CPAP	245	21.4 (18.8–24.2)
Invasive mechanical ventilation	56	4.8 (3.6–6.3)
**Viral codetection** ^¶¶^		
Any viral codetection	286	29.7 (26.4–33.1)
RSV	175	19.3 (16.3–22.5)
Influenza	21	1.9 (1.2–3.0)
Rhinovirus/Enterovirus	64	12.2 (9.3–15.5)
Other viral detections	60	10.8 (8.3–13.9)
Maternal vaccination status***		
No record of maternal vaccination during pregnancy	921	87.5 (85.3–89.5)
Mother vaccinated during pregnancy	144	12.5 (10.5–14.7)
First trimester	62	44.9 (36.1–54.0)
Second trimester	49	33.6 (25.6–42.4)
Third trimester	33	21.5 (15.1–29.1)

**Abbreviations:** BiPAP/CPAP = bilevel positive airway pressure/continuous positive airway pressure; ICU = intensive care unit; RSV = respiratory syncytial virus.

* Excluding birth hospitalizations. A birth hospitalization was defined as the hospitalization during which the infant was born.

^†^ Selected counties in California, Colorado, Connecticut, Georgia, Maryland, Michigan, Minnesota, New Mexico, New York, Oregon, Tennessee, and Utah.

^§ ^Data are from a weighted sample of hospitalized children with completed medical record abstractions. Sample sizes presented are unweighted with weighted percentages.

^¶ ^Persons of Hispanic or Latino (Hispanic) origin might be of any race but are categorized as Hispanic; all racial groups are non-Hispanic. Persons of all other races include non-Hispanic American Indian or Alaska Native and multiracial persons. If ethnicity was unknown, non-Hispanic ethnicity was assumed.

** Defined as one or more of the following conditions: blood disorder, cardiovascular disease including congenital heart disease, chronic lung disease of prematurity/bronchopulmonary dysplasia, asthma/reactive airway disease or airway abnormality, chronic metabolic disease, gastrointestinal disease, genetic disorder, immunosuppressive condition, neurologic disorders, prematurity, renal disease, or other underlying condition.

^†† ^For infants aged <6 months, chronic lung disease includes bronchopulmonary dysplasia and chronic lung disease of prematurity but does not include asthma or reactive airway disease.

^§§ ^COVID-19–related signs and symptoms included respiratory conditions (congestion/runny nose, cough, nausea/vomiting, rash, seizures, shortness of breath/respiratory distress, upper respiratory infection, and wheezing) and non-respiratory conditions (apnea, conjunctivitis, cyanosis, decreased vocalization/stridor, dehydration, diarrhea, fever, hypothermia, inability to eat/poor feeding, and lethargy). Signs and symptoms data were abstracted from medical charts and might be incomplete.

^¶¶ ^Results reported among infants who received testing (as opposed to all hospitalized infants). Because of differing testing practices, denominators differed among the viral respiratory pathogens: 999 infants for any additional virus; 979 infants for RSV, 992 for influenza (influenza A, influenza B, and influenza not subtyped), 521 for rhinovirus/enterovirus, and 523 for other viruses (adenovirus, human metapneumovirus, parainfluenza 1, parainfluenza 2, parainfluenza 3, parainfluenza 4, and other non–SARS-CoV-2 coronaviruses).

*** Maternal vaccination is defined as receipt of COVID-19 vaccine during the pregnancy of the infant hospitalized. A total of 83 (7.4%) infants had unknown maternal vaccination status and were excluded from maternal vaccination status. Proportions presented are calculated with those with known vaccination status (1,065) as the denominator.

### Characteristics of Infant COVID-19–Associated Hospitalizations

The median age at hospital admission for the 1,148 infants for whom clinical information was reviewed was 64 days (IQR = 28–121 days) (Table). A total of 260 (22.1%) infants were admitted to an intensive care unit (ICU), and nine (0.8%) died in hospital; 245 (21.4%) received high-flow nasal cannula or bilevel positive airway pressure/continuous positive airway pressure (BiPAP/CPAP) oxygen support, and 56 (4.8%) received mechanical ventilation. Approximately one quarter (290; 24.0%) of infants had one or more underlying medical condition; the most common conditions identified were prematurity*** (196; 17.1%), cardiovascular disease (74; 6.6%), chronic lung disease^†††^ (58; 4.7%), and neurologic disorders (35; 3.3%). Among 999 (87.1%) hospitalized infants who were tested for additional viruses, at least one other virus was detected among 286 (29.7%), including 19.3% (175 of 979) who received a positive respiratory syncytial virus test result, 12.2% (64 of 521) who received a positive rhinovirus/enterovirus test result, and 1.9% (21 of 992) who received a positive influenza test result. Among 233 (89.8%) of 260 young infants admitted to an ICU who were tested for additional pathogens, an additional virus was detected among 97 (41.2%), including 56 (25.1%) with respiratory syncytial virus detected.

## Discussion

Infants aged <6 months represent one of the population groups most severely affected by COVID-19. During October 2023–April 2024, COVID-19 hospitalization rates among young infants were higher than rates among any other age group except adults aged ≥75 years and were comparable to hospitalization rates in adults aged 65–74 years. The percentage of infants hospitalized with COVID-19 whose mothers had been vaccinated during pregnancy decreased from 17.6% during October 2022–September 2023 to <5% during October 2023–April 2024. Outcomes among hospitalized infants were often serious; excluding birth hospitalizations, approximately one in five young infants hospitalized with COVID-19 required ICU admission, nearly one in 20 required mechanical ventilation, and nine infants died during their COVID-19–associated hospitalization.

Infants aged <6 months are too young to be vaccinated against COVID-19 and generally lack immunity acquired from previous SARS-CoV-2 exposure. To protect young infants from severe COVID-19–associated outcomes, prevention should focus on vaccination of pregnant persons, which protects infants through transplacental transfer of antibodies, and nonpharmaceutical interventions, such as hand hygiene and avoiding exposure to persons with respiratory illness signs and symptoms (*5*,*7*). Maternal vaccination during pregnancy has been shown to be safe and effective in protecting young infants from COVID-19 hospitalization (*3*–*5*); COVID-19 vaccination is recommended by CDC for all persons aged ≥6 months, including those who are pregnant (*8*,*9*). Findings from this analysis are consistent with other evidence demonstrating low COVID-19 vaccine coverage among pregnant persons (*5*), including a 2023 survey of pregnant persons that found that nearly one quarter (24.7%) received a COVID-19 vaccination during pregnancy (*10*). High rates of COVID-19–associated hospitalization among young infants reflect the ongoing vulnerability of this population to severe COVID-19–associated outcomes and indicate an urgent need to improve COVID-19 vaccination coverage among pregnant persons to protect vulnerable infants.

### Limitations

The findings in this report are subject to at least four limitations. First, maternal vaccination information in immunization information systems might not be complete, and misclassification might have occurred. Second, population estimates for infants aged <6 months were not available; rates were calculated using 50% of the population of infants aged <1 year and might not be accurate. Third, COVID-NET relies on clinician-driven testing, and not all hospitalized infants might have been tested for SARS-CoV-2, which might result in underascertainment of COVID-19–associated hospitalizations. Finally, the COVID-NET catchment areas include approximately 10% of the U.S. population, and findings might not be nationally generalizable.

### Implications for Public Health Practice

Infants aged <6 months, who are not yet eligible to receive COVID-19 vaccine, continue to be hospitalized for COVID-19 at higher rates than all age groups except adults aged ≥75 years and at rates comparable to hospitalization rates for adults aged 65–74 years, and severe outcomes were common. During the 2023–24 respiratory virus season, mothers of <5% of infants hospitalized for COVID-19 were vaccinated during pregnancy. To protect pregnant persons and infants too young to be vaccinated, and in the setting of low maternal COVID-19 vaccination coverage, prevention should focus on ensuring that pregnant persons receive recommended COVID-19 vaccines, as well as follow recommendations such as hand hygiene for COVID-19 prevention and newborn care.

## Footnotes

* COVID-NET sites contributing data to this analysis include selected counties in California, Colorado, Connecticut, Georgia, Maryland, Michigan, Minnesota, New Mexico, New York, Oregon, Tennessee, and Utah, representing approximately 10% of the U.S. population.

† Clinical data are complete through April 2024. October–April has been historically defined as the respiratory virus season, although COVID-19 circulates year-round. For October 2021–September 2022 and October 2022–September 2023, 34.9% and 23.6% of pediatric cases, respectively, were reported during the May–September period.

§ Rates are calculated using the National Center for Health Statistics’ unbridged-race postcensal population estimates for the counties or county equivalents included in surveillance. Because population estimates are available in 1-year age increments, population denominators for hospitalization rates among infants aged <6 months were calculated as 50% of the population estimate for infants aged <1 year.

¶ COVID-19–related signs and symptoms included respiratory conditions (congestion/runny nose, cough, nausea/vomiting, rash, seizures, shortness of breath/respiratory distress, upper respiratory infection, and wheezing) and nonrespiratory conditions (apnea, conjunctivitis, diarrhea, cyanosis, decreased vocalization/stridor, dehydration, fever, hypothermia, inability to eat/poor feeding, and lethargy). Signs and symptoms data were abstracted from medical charts and might be incomplete.

** Defined as one or more of the following conditions: blood disorder, cardiovascular disease including congenital heart disease, chronic lung disease of prematurity/bronchopulmonary dysplasia, asthma/reactive airway disease or airway abnormality, chronic metabolic disease, gastrointestinal disease, genetic disorder, immunosuppressive condition, neurologic disorders, prematurity, renal disease, or other underlying condition.

†† During October 2022–April 2024, some sites chose to complete all charts for pediatric patients; the remainder did a random sample. Random numbers (1–100) were generated and assigned to each patient to produce random samples for medical record abstraction stratified by site, age group, and month. Unweighted case counts and weighted percentages that better represent the hospitalized population of the catchment area are presented for sampled data. Percentages were weighted to account for probability of selection for sampled patients.

§§ A birth hospitalization was defined as the hospitalization during which the infant was born.

¶¶ 45 C.F.R. part 46.102(l)(2), 21 C.F.R. part 56; 42 U.S.C. Sect. 241(d); 5 U.S.C. Sect. 552a; 44 U.S.C. Sect. 3501 et seq.

*** Prematurity was defined as gestational age at birth of <37 weeks.

††† For infants aged <6 months, chronic lung disease includes bronchopulmonary dysplasia and chronic lung disease of prematurity. Asthma or reactive airway disease are not included.
